# Concurrent treatment with transarterial immunoembolization of hepatic metastases and systemic immune checkpoint inhibitors to overcome immune evasion in patients with metastatic uveal melanoma

**DOI:** 10.1007/s00262-025-04124-x

**Published:** 2025-07-15

**Authors:** Natalie J. Miller, Sharon W. Kwan, Jacob B. Leary, Daniel S. Hippe, William McCamy, Joshua R. Veatch, Evan T. Hall, Wayne L. Monsky, Shailender Bhatia

**Affiliations:** 1 University of Washington, Seattle, WA USA; 2 Fred Hutchinson Cancer Center, Seattle, WA USA; 3 Colorado Permanente Medical Group, Denver, CO USA

**Keywords:** Uveal melanoma, Choroidal melanoma, Immunoembolization, GM-CSF, Checkpoint blockade

## Abstract

**Background:**

Metastatic uveal melanoma (mUM) is an uncommon melanoma subtype, poorly immunogenic with low objective response rates (ORR) to immune checkpoint inhibitors (ICI). Liver-directed therapies (LDT) are commonly used given the strong predilection for hepatic metastases. Transarterial immunoembolization (TAIE) with granulocyte–macrophage colony stimulating factor (GM-CSF) can potentially synergize with concurrent systemic ICI to overcome immune evasion.

**Methods:**

This single-center, retrospective study includes mUM patients with liver-predominant metastases who received TAIE, with/without concurrent systemic ICI (≤ 3 months before/during TAIE). Endpoints included ORR, progression-free survival (PFS), overall survival (OS), and adverse events (AEs).

**Results:**

Between 2016 and 2023, 18 mUM patients (median age 64 years) received TAIE (median 4 procedures/patient). Fourteen patients (78%) received concurrent ICI. ORR was 17% (3/18), all in patients receiving ICI, with partial responses lasting 4.2, 35 + and 46 months. Disease control rate (stable disease or better) was 56% (10/18). Median time to next systemic therapy or death was 19.5 months (range 1.6- 46). Median PFS and OS from first TAIE treatment were 4.9 months (range 0.7–46) and 35 months (range 1.7- 46). Immune-related AEs (IRAE) during concurrent therapy occurred in seven of 10 patients receiving anti-CTLA-4/PD-1 combination, including hepatitis (n = 5; grade 2 in 1, grade 3 in 4). Four of seven patients resumed anti-PD-1 monotherapy without recurrent IRAE.

**Conclusions:**

Concurrent LDT with GM-CSF TAIE and ICI, including anti-CTLA-4/PD-1 combination, is feasible, safe, and can lead to sustained clinical benefit in a subset of mUM patients. OS with this combination compares favorably to published outcomes for systemic therapy or LDT alone.

**Supplementary Information:**

The online version contains supplementary material available at 10.1007/s00262-025-04124-x.

## Introduction

Uveal melanoma is a rare malignancy that arises from melanocytes within the uveal tract of the eye (including choroid, ciliary body, and iris) and behaves distinctly from cutaneous melanoma. There are approximately 2,000 new cases per year in the USA [1]. Local therapy most commonly consists of radiation (proton vs. photon) or enucleation for larger tumors and is highly effective with infrequent local recurrences. Clinically obvious metastatic disease at time of primary diagnosis is rare (< 3%) [2], but approximately half of patients eventually develop metastatic disease, sometimes decades later highlighting the characteristic dormancy of subclinical metastases. There is a unique predilection for hepatic dissemination with liver being the most common site of metastasis (93%); other sites of metastasis include lung (24%), bones (16%), and soft tissues (11%) [3]. Unfortunately, existing treatments for metastatic uveal melanoma (mUM) have limited efficacy and historical outcomes remain poor, with reported median progression-free survival (PFS) of 3.3 months and overall survival (OS) of 10.2 months in a recent large meta-analysis of patients treated with a variety of modalities [4].

Since most patients have liver-predominant metastases, treatment options for mUM commonly include liver-directed locoregional therapies such as surgery, tumor ablation, and/or hepatic arterial approaches. A broad array of systemic therapies has been investigated such as tyrosine-kinase inhibitors, MEK1/2 inhibitors, cytotoxic chemotherapy (i.e., dacarbazine, temozolomide), epigenetic modifiers, and immune checkpoint inhibitors (ICI). A meta-analysis of 29 clinical trials treating 912 mUM patients evaluated efficacy of these different treatment modalities and found a slight improvement in outcomes for patients who received liver-directed therapies (LDT; median PFS of 5.2 months [95% CI 4.3–5.9], median OS of 14.6 months [95% CI 12.6–17.5]) compared to any systemic therapy alone (median PFS 2.8 months [95% CI 2.7—2.9], median OS 9.3 months [95% CI 8.4–10.1]) [4]. More recently, percutaneous intra-arterial hepatic perfusion (PHP) with melphalan was FDA approved based on results from the FOCUS Phase 3 trial, demonstrating a 35.2% ORR (8% CR) and median PFS > 9 months in carefully selected patients with excellent performance status with small burden of hepatic-only metastasis [5]. However, this complex treatment procedure requires significant infrastructure for implementation and can have serious, life-threatening toxicities. Of note, there is a sparsity of data regarding the combination of liver-directed and systemic therapies.

Trying to mirror the successes against cutaneous melanoma, much effort has been aimed at harnessing the immune system to target UM. Unfortunately, many immunologic barriers exist in this unique malignancy [6]. UM cells have low mutational burden [7], making them less likely to be recognized by the immune system, and often lack expression of Programmed cell death ligand-1 (PD-L1), a biomarker of immunogenicity [8]. Moreover, as the eye is an immune-privileged site, immune infiltration into primary tumors is rare; mUM predominantly develops within the immunosuppressive hepatic microenvironment. Specifically, M2 macrophages are predominant within both the eye and liver, suppressing cytotoxic CD8 T cells both directly through secretion of immunosuppressive cytokines (such as IL-10), and indirectly through stimulation of regulatory T cells (Tregs) [9, 10]. High concentrations of TGF-β and macrophage migration inhibitor factor (MIF) also act to suppress natural killer (NK) cell cytotoxicity at both sites [11]. Hence, it is not completely surprising that responses to ICI have been disappointing, especially in contrast to cutaneous melanoma, with ORR < 5% to agents blocking the PD-(L)1 axis alone [12], 0–8% to anti-Cytotoxic T-lymphocyte associated protein 4 (CTLA-4) alone [13–15], and 11.5–18% to anti-PD-1 + CTLA-4 [16–18]. Recently, tebentafusp, a bi-specific T cell engager that can redirect bystander CD3 + T cells to gp100-expressing UM cancer cells, became the first FDA-approved agent for mUM. However, its applicability is limited to < 50% of patients with HLA-A*02:01 genotype and efficacy is modest with an ORR of only 9% and a median OS of 21.6 months [19, 20].

Hepatic transarterial immunoembolization (TAIE) with the cytokine granulocyte–macrophage colony stimulating factor (GM-CSF) has been proposed as a potential strategy to overcome local immunosuppression in the hepatic microenvironment. GM-CSF can attract immune cells to tumors, activate macrophage and dendritic cells, and enhance tumor-antigen presentation— features that have resulted in its incorporation into oncolytic viruses and anti-cancer vaccines [21]. In a Phase 1 study of TAIE, 34 patients with liver-predominant mUM underwent a median of 6 treatments in cohorts of escalating GM-CSF dose [22]. The treatment was well-tolerated, with reported ORR of 32%, potentially improved OS compared to chemoembolization, and superior outcomes among patients receiving > 1500 mcg GM-CSF. A subsequent randomized Phase II trial comparing TAIE with bland embolization demonstrated improved ORR (21.1% vs. 16.7%) and OS (21.5 vs. 17.2 months) among patients receiving GM-CSF as well as a correlation between post-treatment IL-6 and IL-8 levels and systemic PFS [23], supporting the hypothesis that successful activation of the immune system is possible against this poorly immunogenic disease.

We hypothesized that a combination of liver-directed and systemic immunotherapies may enhance innate and adaptive immune responses needed to achieve anti-tumor benefit. We therefore treated patients at our center with TAIE, with concurrent immunotherapy when feasible. Here, we report our retrospective analysis of efficacy and safety in a cohort of 18 patients with mUM treated with TAIE, 14 (78%) of whom received concurrent ICI.

## Methods

### Study design and participants

This single-center, retrospective study included data from patients diagnosed with mUM who underwent TAIE at the University of Washington and Fred Hutchinson Cancer Center between 2016 and 2023. Data were collected through January 8, 2024. This study was approved by the local Institutional Review Board and was conducted in accordance with the Declaration of Helsinki provisions. Patients eligible for TAIE had liver-predominant metastatic disease as identified by radiographic evaluation, typically abdominal MRI and CT Chest. Patients underwent at least one treatment of TAIE to hepatic metastases, with or without systemic immune checkpoint inhibitors (ICIs). For analysis purposes, concurrent treatment was defined as receiving a dose of ICI within 3 months before initial TAIE treatment and/or during treatment with TAIE.

### Transarterial immunoembolization protocol

**Fig. 1:**
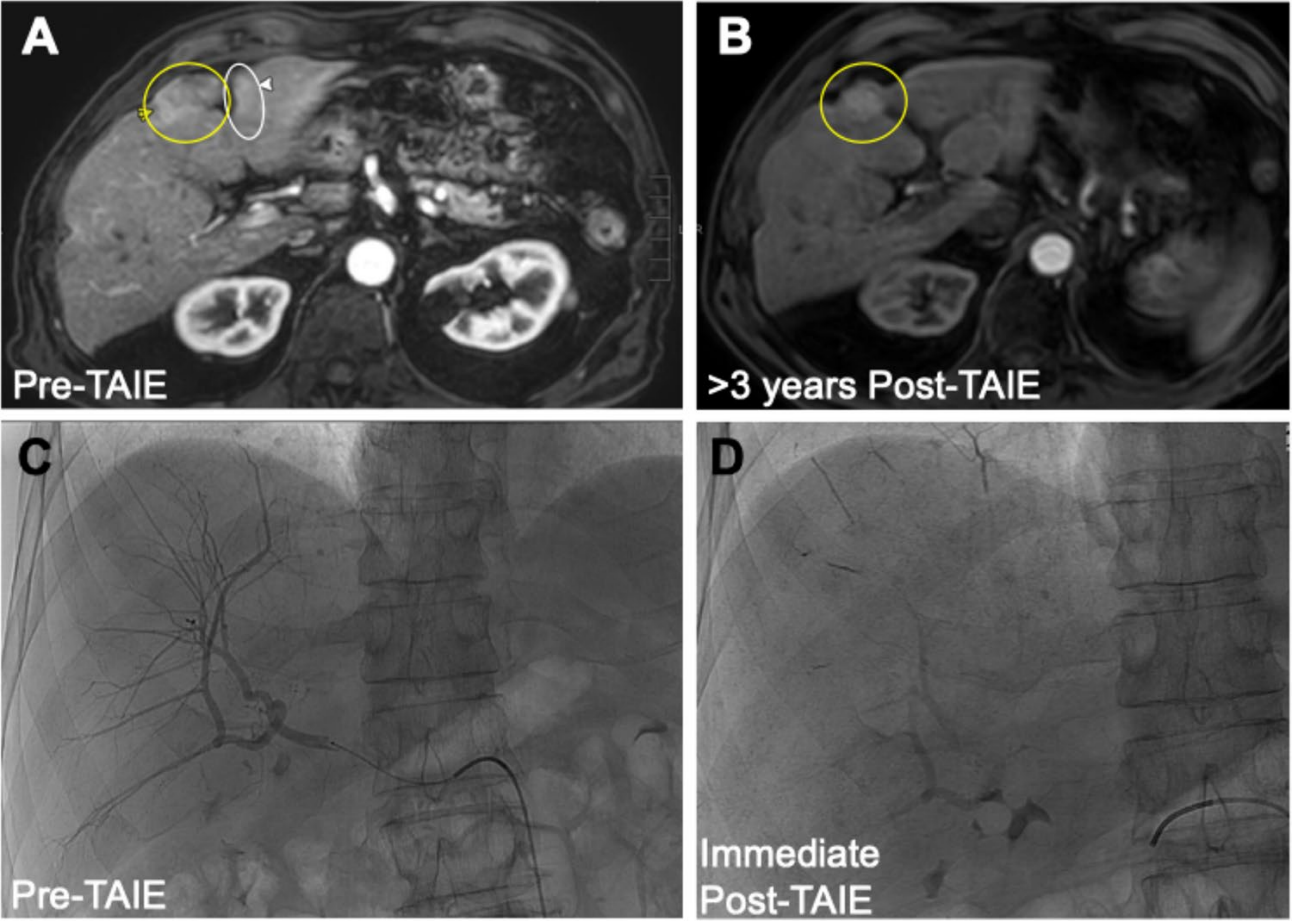
Transarterial hepatic embolization approach leads to longstanding partial response of hepatic metastases in patient treated with TAIE + nivolumab. **A** Pre-treatment MRI in arterial phase of enhancement. Right and left lobe 2.3 cm and 1.4 cm enhancing masses (yellow and white arrow-heads and circles and arrow-heads, respectively). **B** Post treatment MRI in arterial phase of enhancement demonstrates continued response at greater than 3 years post-initial TAIE; right lobe lesion is smaller and the left lobe lesion is not seen. **C** TAIE. Pre-treatment intraprocedural angiogram of right lobe demonstrating multifocal, poorly visualized, small, and punctate areas of tumor blush. **D** Post-TAIE fluoroscopic image from same day with opacification from infused GM-CSF of liver parenchyma and punctate and small foci of intra-tumoral accumulation, scattered in the right lobe and segment 4 as well as perfused vessels

The TAIE protocol included conscious sedation of the patient and local anesthesia, followed by catheterization of the common femoral artery and arteriography to access selected branch of the hepatic arterial system to reach target hepatic metastases. Immunoembolization mixture of GM-CSF (sargramostim; Sanofi-Aventis U.S. LLC) at a total anticipated dose of 2000 mcg in combination with 10 cc Lipiodol (Guerbet LLC) was slowly infused into the selected artery, followed by slow infusion of gelfoam slurry suspended in contrast material. Both were infused to point of near stasis, i.e., sluggish, pulsatile arterial flow with a “pruned-tree” appearance of branches, without reflux or non-target embolization. Post-embolization arteriography was performed to ensure near stasis of supplying arterial branches and lack of angiographic tumor blush, which were expected endpoints (Fig. 1). All catheters were removed when hemostasis was achieved. This procedure was repeated in an alternating right/left lobar arterial infusion approximately every 4 weeks for typically up to 10 treatments (except one patient who underwent 12 procedures total). When feasible, patients who completed 10 treatments of TAIE received transarterial chemoembolization (TACE) (*n* = 3; typically two treatments of alternating lobar doxorubicin and two treatments of 1,3-bis(2-chloroethyl)-1-nitrosourea (BCNU), though one of these three patients did not receive the last treatment of BCNU due to starting subsequent systemic therapy).

### Immune checkpoint inhibitors

ICI agents included the FDA-approved PD-1 inhibitors such as pembrolizumab or nivolumab administered as monotherapy or the combination of CTLA-4 inhibitor, ipilimumab (1 mg/kg IV) plus nivolumab (3 mg/kg IV) administered at standard FDA-approved dosing schedules. The administration of ICI agents was adjusted as needed to accommodate the scheduled TAIE procedures, given the logistical complexities related to scheduling the latter. ICI administration was typically planned to precede the TAIE procedures, to spare the patients from traveling to the infusion center while recovering from TAIE, and to mitigate theoretical concerns about inducing immune-mediated hepatotoxicity if ICI is administered soon after the expected hepatic injury from TAIE.

### Assessments

Clinical data retrieved from electronic medical records included patient demographics such as age, race, sex, clinicopathologic details regarding primary uveal melanoma (date of diagnosis, stage, primary treatment modality, and gene expression profile category [DecisionDx-UM by Castle Biosciences]), date of documented metastatic disease, prior hepatic and/or systemic treatments, sites of metastasis at the time of TAIE, percentage of liver involvement (contrast enhanced arterial and venous phase CT and or MRI obtained within 6 weeks from each TAIE cycle was used by Interventional Radiologist to make three measurements [x, y, and z planes] in axial and coronal images to estimate liver and tumor volume with Picture Archiving and Communication System [PACS] measurement tools), Eastern Cooperative Oncology Group (ECOG) performance status at time of first TAIE, number of TAIE treatments, use of concurrent immunotherapy (type and timing in relation to initiation of TAIE), subsequent liver-directed and/or systemic therapies, and adverse events.

Tumor response was assessed approximately every 3 months by contrast-enhanced cross-sectional imaging, typically an abdominal MRI. Safety associated with TAIE and/or ICI therapy was evaluated by adverse event (AE) reporting, including immune-related adverse events (IRAE), graded by Common Terminology for Adverse Events (CTCAE) version 5.0 criteria. For IRAE, the CTCAE grade, use of steroids and/or any additional immunosuppressive agents, resumption of ICI and further IRAE after resumption were collected.

### Statistical analysis

All mUM patients who received TAIE at our institution during the study period were included in this retrospective analysis. No a priori power calculations were performed, as the study was descriptive and based on the available patient population. Descriptive statistics were used to determine median age at first TAIE and median number of TAIE treatments. Investigator assessment of objective response status was done following criteria within RECIST Version 1.1 [24]. The best overall response of hepatic metastases from the start of TAIE was also assessed. PFS was calculated from the date of initiation of TAIE to the date of radiographic progression, death, or last follow-up. OS was measured from the date of initiation of TAIE to the date of death or last follow-up. Time to next systemic therapy or death was defined from the date of initiation of TAIE to the date of next treatment or date of death; patients alive without a subsequent systemic therapy were censored at last follow-up. The Kaplan–Meier method was used to calculate survival curves, median time to next systemic therapy curve, and corresponding 95% confidence intervals. There were no missing values recorded for any variable reported.

## Results

### Patient characteristics

A total of 18 patients with mUM underwent TAIE at our center between 2016 and 2023 and were included in this analysis. Demographic and clinical data from these patients are summarized in Table 1. Patients selected for TAIE had liver-predominant metastases at baseline, good performance status (ECOG 0–1), and adequate hepatic function. Data were collected through January 8th, 2024. The median time from primary uveal melanoma diagnosis to initiation of TAIE was 22 months (range 9.0–58 months), with a median time from diagnosis of mUM to initiation of TAIE of 2.7 months (range 17 days–14 months). Five patients had received therapy for metastatic disease prior to TAIE; one patient received LDT only (radiofrequency ablation [RFA]), two patients received systemic ICI only (ipilimumab and nivolumab), and two patients received combination of LDT (TACE, *n* = 1; RFA, *n* = 1) with systemic ICI. All patients had developed progressive disease prior to initiation of TAIE.

**Table 1:** Patient demographics and baseline characteristics (n = 18)

Median age at first TAIE, years (range)	64.1 (46.3–80.6)
Sex, n (%)	
Male	8 (44.4)
Female	10 (55.6)
Race, n	
Caucasian	14
Other	4
AJCC Prognostic Stage Group at primary diagnosis, n	
Unknown	1
Stage I	1
Stage IIA	5
Stage IIB	3
Stage IIIA	4
Stage IIIB	4
Stage IIIC	0
Stage IV	0
DecisionDx-UM (Castle) category, n	Not available = 4
1A	2
1B	1
2	11
ECOG, n	
0	14
1	4
Liver involvement, n	
< 20%	16
> 20%	2
Extrahepatic disease at first TAIE, n	3
Bone	1
Bone + Abdominal wall	1
Abdominal wall	1
Prior therapy, n	5
Liver-directed only Liver-directed + ICI	1 2
ICI only	2
Concurrent immunotherapy, n	14
Anti-PD-(L)1 monotherapy	4
Anti-PD-1/CTLA-4 combination	10

In total, 93 TAIE treatments were performed with a median number of treatments per patient of 4 (range: 1–12). Median follow-up time was 24 months (range: 1.7–54 months). Most patients (14 of 18, 78%) received concurrent ICI with TAIE or within 3 months before starting TAIE. Of these, 10 were treated with combined anti-CTLA-4 and anti-PD-1 therapy and four with anti-PD-1 monotherapy; three of four had previously been treated with anti-CTLA-4 and anti-PD-1 therapy combination but > 3 months prior to TAIE. Anti-PD-1 monotherapy included pembrolizumab or nivolumab. Anti-PD-1/CTLA-4 combination was nivolumab + ipilimumab.

### Treatment-related outcomes

Response data are summarized in Table 2. ORR was 17% (*n* = 3, out of 18), with all three patients experiencing partial response (PR) and receiving concurrent ICI, including anti-CTLA-4 plus anti-PD-1 therapy (*n* = 2) and anti-PD-1 monotherapy (*n* = 1). Duration of response was 4.2 months, 35 months (ongoing), and 46 months in these three patients. Pre- and post-treatment MRI from one patient with a longstanding PR after treatment with TAIE + nivolumab is shown in Fig. 1, along with pre-treatment intraprocedural angiogram demonstrating areas of tumor blush and same day post-TAIE fluoroscopic image with opacification from infused GM-CSF of liver parenchyma and intra-tumoral accumulation.

**Table 2:** Best Radiologic Response of Hepatic Metastases during TAIE

		Concurrent systemic therapy		
	N (%)	α-PD-(L)1	α-PD-(L)1 + CTLA-4	None
CR	0 (0%)	0	0	0
PR	3 (17%)	1	2	0
SD	7 (39%)	3	3	1
PD	8 (44%)	0	5	3

**Fig. 2:**
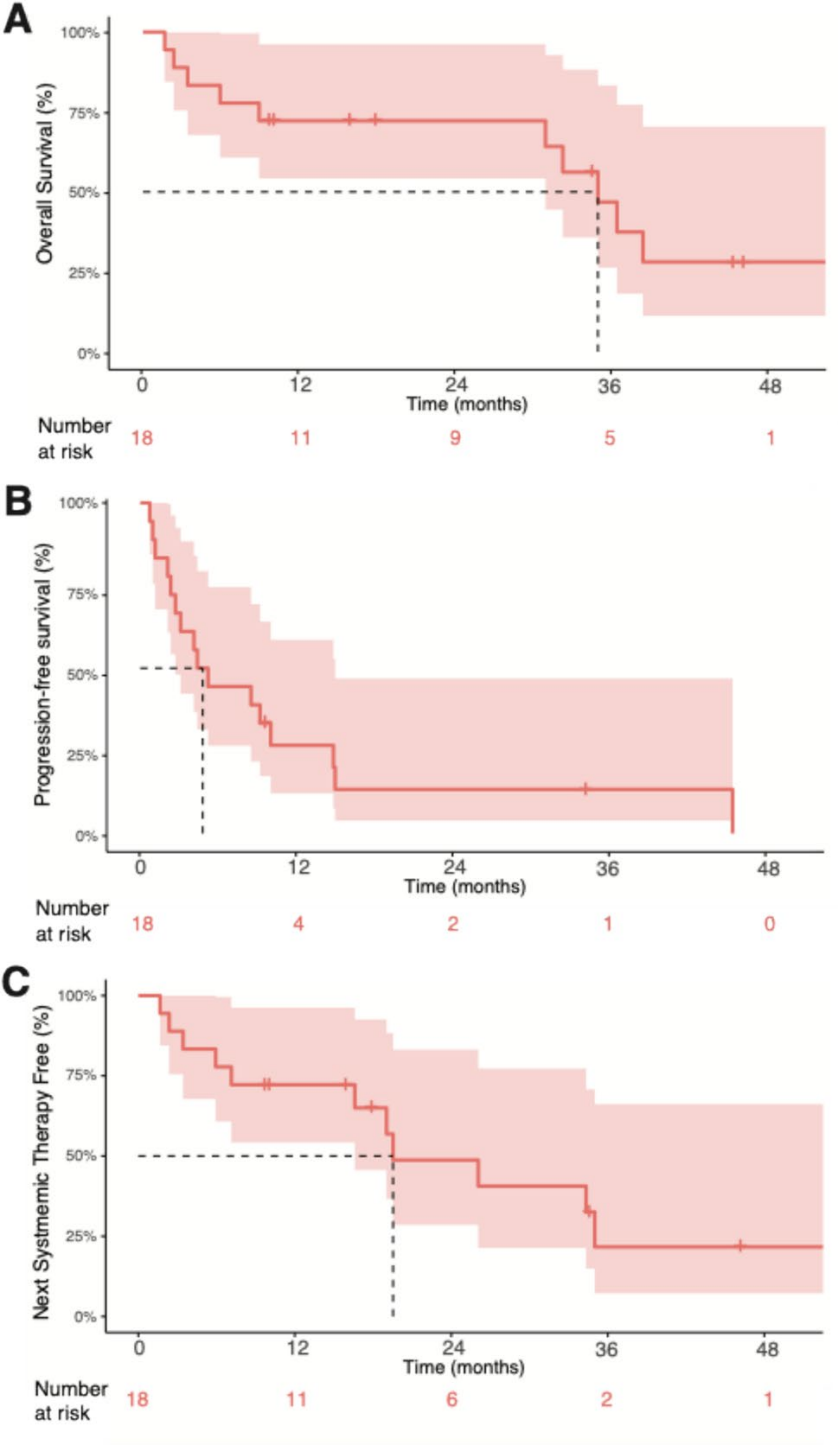
Kaplan–Meier plot of **A)** Progression-free survival **B)** Overall Survival and **C)** Time to Next Systemic Therapy or Death of all patients in cohort from time of initiating TAIE. **ABC** Median values are indicated by dashed line. 95% CI is shaded

An additional seven patients had a best response of stable disease (SD), with a disease control rate of 56% (*n* = 10 of 18 patients); five of these seven patients received concurrent ICI (*n* = 2 with PD-1 monotherapy, *n* = 3 with dual ICI). The median hepatic PFS from the time of first TAIE among all patients was 4.8 months (range: 0.7–46 months; 95% CI 2.7–15.1) as illustrated in Fig. 2. Sixteen of 18 patients had developed PD by the data cutoff date, with two patients having ongoing disease control lasting 35 months (PR) and 9.7 mo (SD). Among all 18 patients, 8 were alive at time of data cutoff. The median OS from time of first TAIE was 35 months (range: 1.7–46 months; 95% CI 31–not reached [NR]; see Fig. 2).

**Fig. 3:**
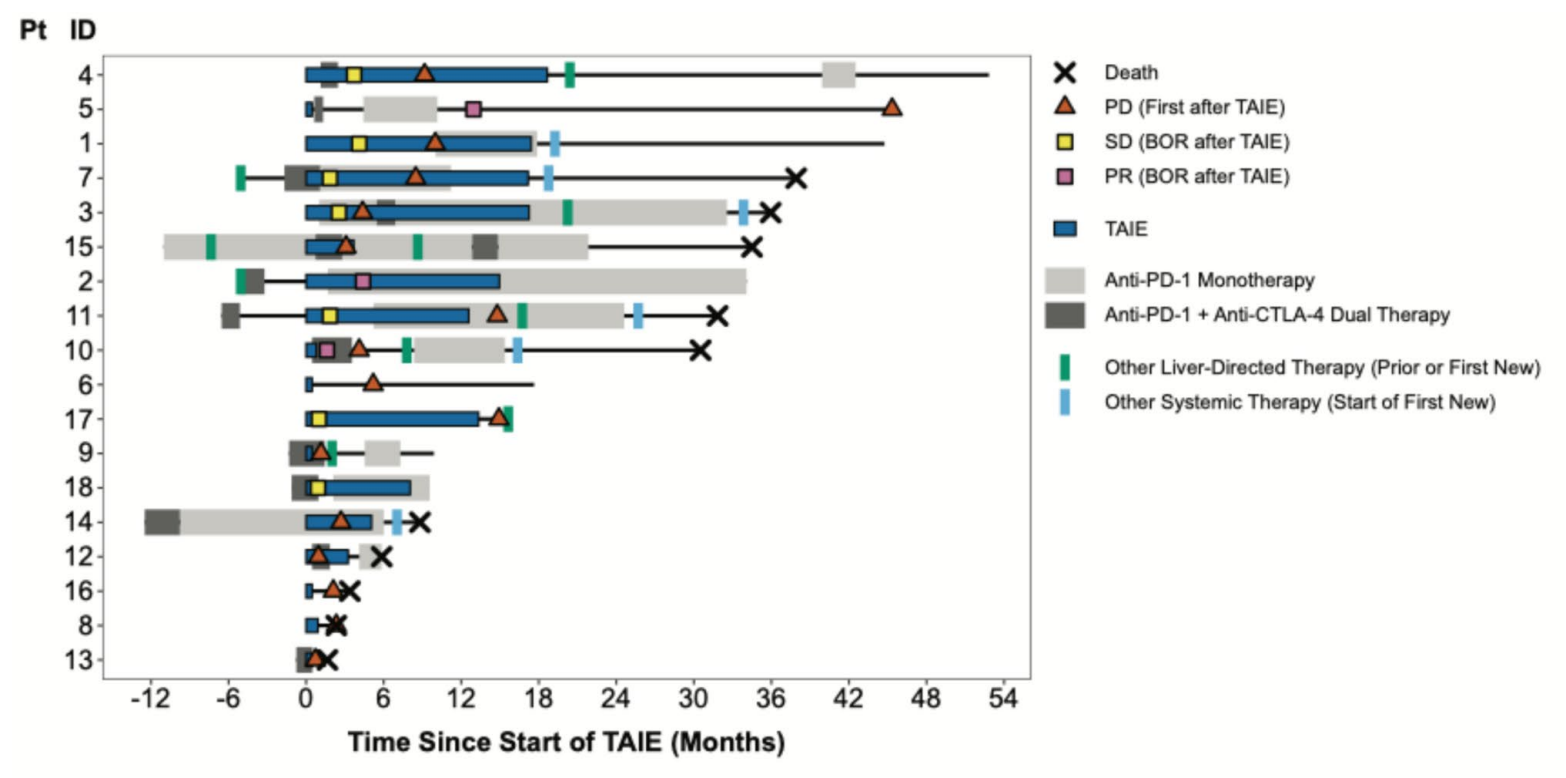
Swimmer Plot of each patient’s individual treatment course. Liver-directed and systemic therapies before, during, and after duration of TAIE (blue box) are depicted. Concurrent ICI is depicted as gray bars, with dark gray during period of concurrent ipilimumab/nivolumab, and light gray during PD-1 monotherapy. Five patients underwent local (green bar) or systemic immunotherapy (gray bar[s]) that started ≥ 3 months prior to first TAIE; Ten patients underwent post-TAIE treatments for progressive disease depicted as green bars (liver-directed, *n* = 7) or blue bars (systemic, *n* = 6). Best response and/or date of first progression after starting TAIE, if applicable, is noted for each patient. Death is depicted as an X, while all other patients were ongoing follow-up at time of data cutoff

In addition, among the entire cohort, the median time to next systemic therapy (*n* = 6) or death (*n* = 5) was 19.5 months (range: 1.6–54 months; 95% CI 17–NR) (Fig. 2). Next systemic therapies included tebentafusp (*n* = 2), trametinib (*n* = 1), and novel investigational agents on a clinical trial (*n* = 3). Seven patients underwent subsequent liver-directed therapies at some point after TAIE ± 2 cycles of doxorubicin and BCNU per protocol; three also received systemic therapy and four underwent LDT only. Next hepatic therapies for these patients included radioembolization (Y-90 × 2, *n* = 4) and chemoembolization (BCNU × 1, *n* = 2; doxorubicin × 4, *n* = 1). Summarized subsequent treatment data for the cohort can be found in Supplemental Table 1. The clinical course for each patient, including preceding therapies, periods of treatment with TAIE + concurrent ICI, and subsequent therapies is illustrated via swimmer plot (Fig. 3).

### Impact of disease burden on outcomes

**Fig. 4:**
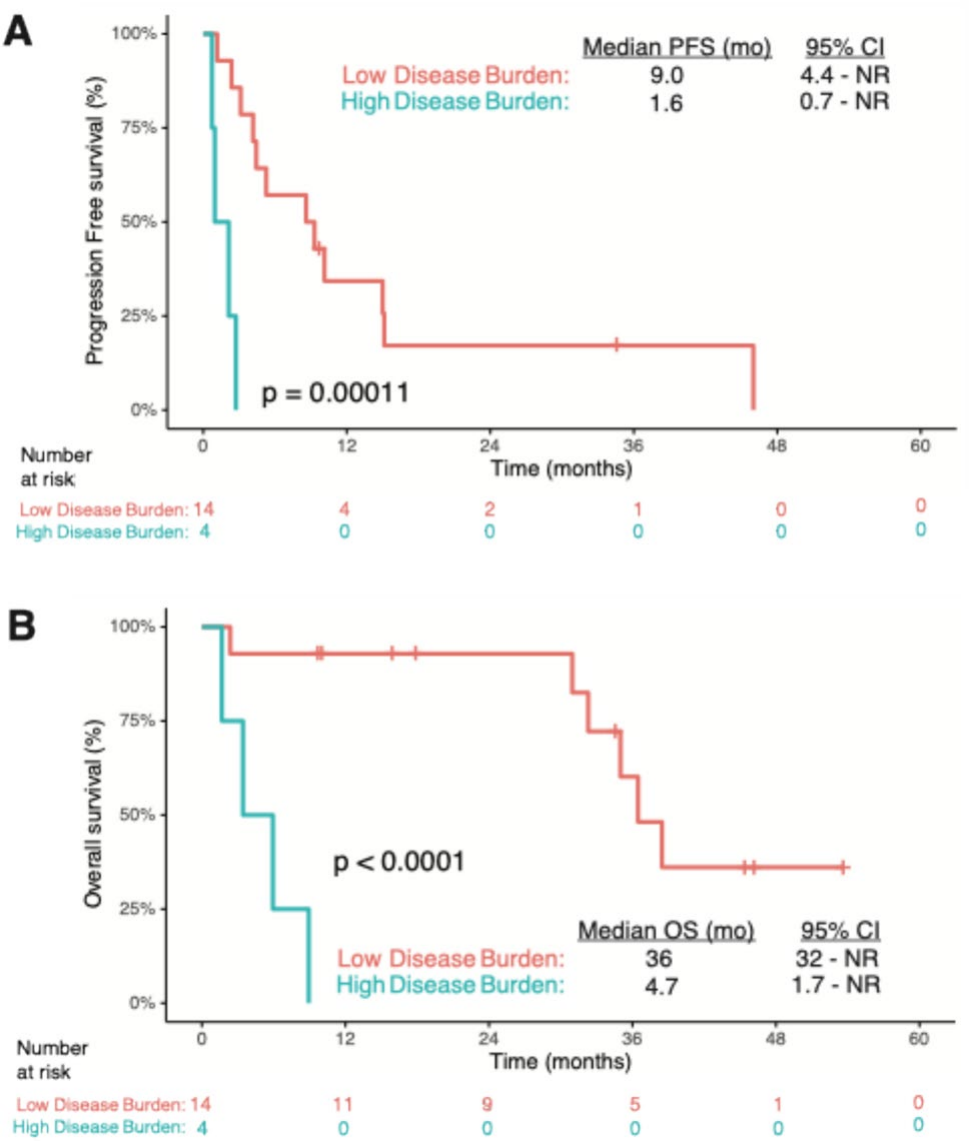
Tumor Burden and Outcomes: Low disease burden is associated with significantly longer PFS and OS compared to patients with high disease burden. Kaplan–Meier plot of **A**) Progression-free survival **B**) Overall Survival for all patients in cohort from time of initiating TAIE, by disease burden of either low (red; < 20% hepatic involvement and no extrahepatic metastases; *n* = 14) or high (blue; > 20% hepatic involvement or extrahepatic metastasis, *n* = 4). **A** **B** Median survival for each group is noted with 95% CI

Four of 18 (22%) patients had extrahepatic metastases (*n* = 3; spine only *n* = 1, peritoneum only *n* = 1, both *n* = 1) and/or greater than 20% involvement of liver prior to TAIE (*n* = 2, approximately 90% and 75%). These patients are represented in Fig. 3 as #14, #16, #12, and #13, respectively. Three of these four patients received concurrent systemic therapy (ipilimumab + nivolumab, *n* = 2; nivolumab monotherapy after ipilimumab > 3 mo prior to TAIE, *n* = 1; Supplemental Table 2). These four patients received fewer treatments with TAIE (median 2.5 compared to median of 7 among patients with < 20% hepatic involvement; *n* = 14). All four patients had disease progression at the first scan after starting TAIE, and two continued TAIE beyond progression. Only one patient received additional local or systemic therapy (single cycle on clinical trial of systemic MEKi); all four ultimately experienced progressive disease leading to death. These four patients had a significantly shorter median PFS and OS than the 14 patients with ≤ 20% hepatic involvement and no extrahepatic metastases prior to TAIE (PFS of 1.6 vs. 9.0 months, *p* = 0.001; OS of 4.7 vs 36 months, *p* < 0.0001; Fig. 4).

### Safety and tolerability of therapy

TAIE alone and TAIE + ICI were generally well-tolerated in this cohort. One patient had transient distributive shock immediately after a TAIE procedure requiring pressors and hydrocortisone in an intensive care setting, from which the patient quickly and fully recovered; this was not felt to be an IRAE. IRAEs were observed in seven of 10 patients receiving combination ipilimumab/nivolumab with concurrent TAIE, 0 of four patients receiving anti-PD-1 monotherapy with concurrent TAIE, and 0 of four patients receiving TAIE monotherapy. These patients are #s 3, 4, 5, 9, 10, 12, and 18 in Fig. 3. IRAE while on concurrent TAIE + ICI are summarized in Table 3. IRAE experienced by patients on ICI only (> 3 months before or after TAIE) were not reported given frequency of such events is well documented in the literature.

**Table 3:** Immune related adverse events on concurrent TAIE + ICI (*n* = 14)

	Any grade N (%)	Grade ≥ 3 N (%)
Any IRAE*	7 (50%)	5 (36%)
Hepatitis**	5 (36%)	4 (29%)
Colitis	2 (14%)	2 (14%)
Pancreatitis	1 (7%)	1 (7%)
Adrenal insufficiency	1 (7%)	1 (7%)
Pneumonitis	1 (7%)	0
Thyroiditis	1 (7%)	0
Rash	2 (14%)	0
Pruritis	2 (14%)	0

*All seven patients received anti-PD-1/CTLA-4 combination

**Four of five patients with hepatitis were treated with steroids; hepatitis resolved in all patients

Hepatitis was the most common IRAE (*n* = 5), with severity ≥ Grade 3 in four of five patients; steroids were given to four of five patients and hepatitis resolved in all patients. Grade ≥ 3 colitis was observed in two patients, felt secondary to ipilimumab, and resolved with steroids (*n* = 1) and/or infliximab (*n* = 1). Six of seven patients who experienced IRAE restarted anti-PD-1 monotherapy, which was well-tolerated in four patients; two patients discontinued anti-PD-1 for a new IRAE in a previously uninvolved organ system. No recurrent hepatitis was noted. We did not see any significant relationship between toxicity and response rate, OS, or PFS between patients with or without IRAE.

## Discussion

In this retrospective study, we share our institutional experience of using hepatic TAIE with GM-CSF in 18 patients with mUM. Our experience demonstrates feasibility of combining TAIE with concurrent systemic ICI (anti-PD-1 ± anti-CTLA-4) in 14 patients. Overall therapy was well-tolerated and safe, with Grade ≥ 3 IRAE in six of 10 patients on triple therapy with TAIE, ipilimumab, and nivolumab that all resolved with standard treatment, and one transient serious AE thought to be related to TAIE only. Disease control rate in our cohort was 56% (10 of 18), with three patients (17%; 3 of 18) experiencing an objective response (PR). Two of three responding patients had benefit that lasted > 34 months without additional therapeutic intervention.

The observed median PFS of 4.8 months is similar to previous studies of hepatic-directed therapies, including the original Phase 1 study of TAIE alone (hepatic PFS of 4.8 mo) [22], the first randomized controlled trial (RCT) of TAIE vs. bland embolization (hepatic PFS 3.9 mo) [23] and meta-analysis including various liver-directed therapies (5.2 mo) [4]. Of note, ORR and PFS may not be the best endpoints to establish benefit of this treatment approach, given the prolonged TAIE treatment course over several months in an alternating lobar fashion and the radiologic changes caused by TAIE itself, such as lipiodol accumulation, post-treatment inflammatory, and perfusion changes, that can render assessment of clinically significant tumor growth challenging. Alternative methods of assessment, such as mRECIST [25], are challenging due to low baseline enhancement of mUM lesions. Indeed, six of 12 patients who experienced disease progression while being treated with TAIE continued treatment given perceived benefit in slowing disease growth and the possibility to direct subsequent treatments to progressing tumors (analogous to intra-tumoral immunotherapy approaches). The median OS in our study was 35 months, which appears favorable as compared to historic median OS of TAIE alone (21.5 months) [23] and another recent study of TAIE + pembrolizumab alone (21.3 month) [26], as well as to patients treated with tebentafusp (21.6 mo) [20]. In addition, data from this study compare favorably to patients treated with PHP with melphalan, which was associated with median OS of 20.5 months [5] or 18.4 mo [27].

Within our patient cohort, we observed a dichotomy into two subgroups based on markedly different outcomes; those with high hepatic burden (> 20%) and/or extrahepatic disease (*n* = 4) who all died within 9 months, and the remaining cohort that on average lived > 3 years post first TAIE. Specifically, the cohort of patients with a lower burden of disease (< 20% hepatic involvement and no extrahepatic disease; *n* = 14) had a significant improvement in both PFS and OS compared to patients with extrahepatic and/or > 20% hepatic involvement by metastasis (*n* = 4). Our findings may reflect biologic differences in disease between these two subgroups, and/or the limitations of this treatment strategy to stimulate clinically meaningful immune responses in the high burden group. It is also plausible that an absence of extrahepatic metastases along with low hepatic burden permits greater number of TAIE cycles (as was observed in this study), and this may allow more time for maturation of immune responses in the treated tumors and hence facilitate better systemic disease control. During the first RCT of TAIE versus bland embolization for mUM, liver involvement > 20% was similarly found to be associated with poorer systemic PFS and OS even upon multivariate analysis [23]. Therefore, it may be prudent to consider disease burden when evaluating patients for treatment with TAIE + ICI or during other investigations of locoregional plus systemic immunotherapy combinations in the future.

Activation of the innate immune system via liver-directed approaches offers a unique and promising opportunity for synergy with systemic ICI in enhancing the adaptive immune response. Several prospective studies are currently underway to evaluate such combinations including the phase II study of ipilimumab and nivolumab with TAIE at Thomas Jefferson University (NCT03472586) and the phase 1/1b study of intrahepatic SD-101, a TLR9 agonist, alone or in combination with ICI (PERIO-01; NCT 04935229). Preliminary studies show encouraging early efficacy signals in patients treated in PERIO-01, with a disease control rate of 81% among patients treated with the optimal dose of 2 mg SD-101 with nivolumab (*n* = 7) [28]. Moreover, there was evidence of increasing CD8 and CD4 T cells and NK cells within hepatic metastases after treatment, along with increased TLR and IFN signaling and Th1 T cell activation by gene expression analysis, suggesting tumor inflammation as a mechanism of response [28]. In addition, novel engineered herpesviruses that have been altered to express GM-CSF such as RP2 have led to early promise in patients with uveal melanoma. Results from a Phase 1 study of RP2 ± nivolumab in patients refractory to standard ICI show an ORR of 33% for RP2 monotherapy and 29.4% for RP2 + nivolumab, with an additional 29.4% of that cohort experiencing stable disease; responses were observed in non-injected lesions [29]. Together these promising early trials along with our findings support strategies that combine local and systemic immune stimulation to overcome the local immunosuppression.

Limitations of our retrospective study include the small sample size (*n* = 18) as well as the heterogeneity of treatment interventions including the number of TAIE treatments (range 1–12), and type and timing of systemic ICI. Strengths include demonstration of feasibility and safety in a real-world setting spanning several years, and the observation of durable responses in a subset of patients, suggesting possible synergy of a liver-directed immunotherapy with systemic ICI in overcoming immune evasion in a cancer type historically considered to be poorly immunogenic. While TAIE requires partnership with Interventional Radiology at a tertiary medical center, it is considerably less toxic than melphalan PHP (which additionally requires cardiac perfusionist support) and less resource-intensive than therapies such as tebentafusp, which requires inpatient admission and close monitoring followed by weekly outpatient infusions; these differences may additionally be appealing to clinicians and patients.

In summary, concurrent liver-directed therapy with GM-CSF TAIE and systemic ICI, including anti-CTLA-4/PD-1 combination, is feasible and safe, and can lead to sustained clinical benefit in a subset of UM patients; patients with < 20% hepatic involvement appeared to benefit the most from this approach. Liver-directed immunotherapy approaches may synergize with systemic immunotherapies to overcome immune evasion in this poorly immunogenic cancer with predilection for hepatic metastases.

## Supplementary Information

Below is the link to the electronic supplementary material.

Supplementary file1 (PDF 39 KB)

## Data Availability

The datasets generated during and/or analysed during the current study are available from the corresponding author on reasonable request.

## Abbreviations

AE: Adverse event(s)
BCNU: 1,3-Bis(2-chloroethyl)-1-nitrosourea
CR: Complete response
CTCAE: Common terminology for adverse events
CTLA-4: Cytotoxic T-lymphocyte associated protein 4
ECOG: Eastern cooperative oncology group
GM-CSF: Granulocyte–macrophage colony stimulating factor
ICI: Immune checkpoint inhibitor(s)
IRAE: Immune-related AE(s)
MUM: Metastatic uveal melanoma
NK: Natural killer
ORR: Objective response rate
OS: Overall survival
PD: Progressive disease
PD-1: Programmed cell death protein 1
PD-L1: Programmed cell death protein ligand 1
PFS: Progression-free survival
PR: Partial response
RCT: Randomized control trial
RFA: Radiofrequency ablation
SD: Stable disease
TACE: Transarterial chemoembolization
TAIE: Transarterial immunoembolization
