# Supplementary material for: Concurrent treatment with transarterial immunoembolization of hepatic metastases and systemic immune checkpoint inhibitors to overcome immune evasion in patients with metastatic uveal melanoma
Source: Cancer Immunol Immunother. 2025 Jul 15;74(8):270. doi: 10.1007/s00262-025-04124-x (PMC12263499; doi:10.1007/s00262-025-04124-x)
Supplement: Supplementary file 1 — Supplementary file1 (PDF 39 KB) [file 262_2025_4124_MOESM1_ESM.pdf]

Supplemental Table 1: Subsequent therapies	
**Type of Therapy**	**n (%)**
Systemic only	3 (17%)
Liver-directed only	4 (22%)
Systemic + Liver-directed	3 (17%)
None – not needed	2 (11%)
None – comfort	6 (33%)

Supplemental Table 2: Characteristics among patients with High vs Low tumor volume		
	Low volume disease (n=14)	High volume disease (n=4)
**Median age at first IE, years (range)**	63 (46-80)	70.5 (49-79)
**Sex, n**		
Male	8	0
Female	6	4
**Race, n**		
Caucasian	11	3
Other	3	1
**AJCC Prognostic Stage Group at primary diagnosis, n**		
unknown	1	0
Stage I	1	0
Stage IIA	4	1
Stage IIB	3	0
Stage IIIA	2	2
Stage IIIB	3	1
Stage IIIC	0	0
Stage IV	0	0
**GEP by Castle, n**	N/A = 3	N/A = 1
1A	2	0
1B	1	0
2	8	3
**ECOG, n**		
0	13	1
1	1	3
**Prior therapy, n**	4	1
Liver-directed only	1	0
Liver-directed + ICI	2	0
ICI only	1	1
**Concurrent immunotherapy, n**	11	3
Anti-PD-(L)1 monotherapy	3	1
Anti-PD-1/CTLA-4 combo	8	2
**Median number of TAIE (range)**	7 (1-12)	2.5 (1-4)
